# Biomechanical testing of different fixation techniques for intraoperative proximal femur fractures: a technical note

**DOI:** 10.1080/23335432.2022.2142159

**Published:** 2022-11-20

**Authors:** Toni Wendler, Benjamin Fischer, Alexander Brand, Martin Weidling, Johannes Fakler, Dirk Zajonz, Georg Osterhoff

**Affiliations:** a ZESBO Centre for Research on Musculoskeletal Systems, Leipzig University, Leipzig, Germany; b Institute of Anatomy, Leipzig University, Leipzig, Germany; c Department of Orthopaedics Trauma and Plastic Surgery, Leipzig University, Leipzig, Germany; d Department of Neurosurgery, Leipzig University, Leipzig, Germany; e Department of Orthopaedic, Trauma and Reconstructive Surgery, Zeisigwaldkliniken Bethanien, Chemnitz, Germany

**Keywords:** Intraoperative proximal femoral fracture, fixation techniques, cerclage band, cerclage wires, lag screw fixation, total hip arthroplasty, biomechanical study

## Abstract

Intraoperative proximal femoral fractures (IPFF) represent a rare but challenging complication of total hip arthroplasties. They usually occur as a longitudinal split. This pilot trial aimed to compare the biomechanical primary stability of different fixation techniques for IPFF. Standardised longitudinal medial split fractures of the proximal femur (type II, Modified Mallory Classification) were created in artificial osteoporotic and non-osteoporotic composite femora after implantation of a cementless femoral stem. Five different fixation techniques were compared: cerclage band, cerclage wiring with one or two wires, and lag screw fixation with one or two lag screws. A quasi-static loading protocol was applied and failure loads were evaluated. The observed median failure loads were 4192N (3982N – 5189N) for one cerclage band, 4450N (3577N – 4927N) for one cerclage wire, 5016N (4175N – 5685N) for two cerclage wires, 6085N (5000N – 8907N) for one lag screw, and 4774N (4509N – 8502N) for two lag screws. Due to the wide range of failure loads within the experimental groups, there were no observable differences between the groups. All fixation techniques provided sufficient primary stability in osteoporotic and non-osteoporotic composite bones. Further cadaveric studies with larger sample sizes may be needed to confirm the results presented here.

## Introduction

With an incidence of 0.18% per year over the entire population, total hip arthroplasty (THA) is one of the most common surgical procedures in OECD (Organisation for Economic Co-operation and Development) member states (OECD 2019). Intraoperative proximal femoral fractures (IPFF) represent a rare but challenging complication of THA. They usually occur as a longitudinal split of the proximal femur. Incidence rates of around 1.0% to 3.2% have been reported even in experienced hands (Masri et al. 2004; Watts et al. 2015; Zhao et al. 2017; Liu et al. 2019). Due to generally lower bone mass density, geriatric patients suffer from IPFF more frequently. Furthermore, IPFF are significantly more likely in revision surgery than in primary THA (Berry 1999; Della Rocca et al. 2011). One reason for this is that IPFF in primary THA occur predominantly during stem insertion, whereas in the context of more extensive revision surgery, several scenarios exist that can lead to the initiation of IPFF. (Berry 1999).

Longer recovery time and prolonged immobilisation associated with IPFF can result in thromboembolic complications and increased mortality (Capone et al. 2017,2017; Horner et al. 2019). Therefore, direct stabilisation of the fracture during primary intervention is of great importance to the long-term success of THA (Berend et al. 2004). Due to good clinical outcomes (Berend et al. 2004) and high primary stability (McCulloch et al. 2012) cerclage bands, wires or cables are chosen in most cases (Berend et al. 2004; Angelini and Battiato 2015). The main objective is to increase primary stability allowing immediate mobilisation, fracture healing, and ingrowth of the femoral stem.

While cerclage bands are considered the gold standard by some researchers, there is controversy regarding their influence on periosteal blood flow (Lenz et al. 2012a; Apivatthakakul et al. 2013). Alternative treatment options include cerclage cables and wires, lag screw fixation, or – much more invasive – plate fixation. Lag screw fixation is characterised by being a minimally invasive approach and not interfering with the blood supply to the periosteum. However, no comparative biomechanical studies investigating lag screw versus cerclage fixation of IPFF were already conducted. Hence, this pilot trial aimed to compare the biomechanical primary stability of various IPFF fixation techniques.

## Materials and methods

### Specimen preparation

Five different fixation techniques were used to treat three fractured artificial composite femora (Sawbones USA, Pacific Research Laboratories, Vashon, Washington, USA) each, from which two femora were of non-osteoporotic (4th Gen., Composite, 17 PCF Solid Foam Core, Medium) and one of osteoporotic (4th Gen., Composite, 12.5 PCF Cellular Foam) bone quality. Three specimens were chosen based on the methodology of Frisch et al. (Frisch et al. 2015).

**Figure 1.**
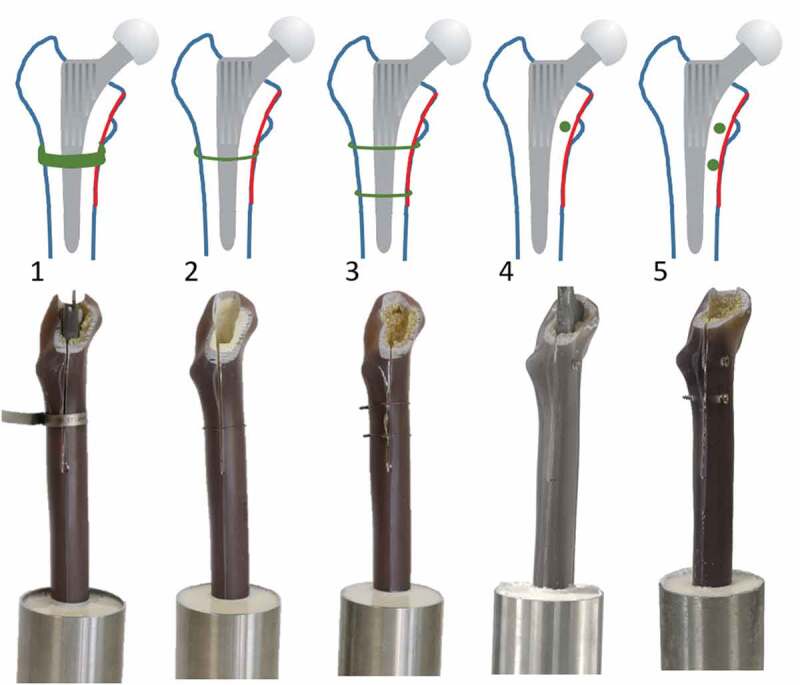
Treatment variants from left to right: (1) cerclage band, (2) simple cerclage, (3) double cerclage, (4) lag screw fixation with one screw, (5) lag screw fixation with two screws. The standardised fracture is shown as red line.

First, a standard Spotorno femoral stem (CBC Evolution, size 8, Mathys AG Bettlach, Bettlach, Switzerland) was implanted by an experienced senior surgeon (DZ). Standardised intraoperative fractures (type II, Modified Mallory Classification) (Ferbert et al. 2020) were created propagating medially along the femoral shaft using an oscillating saw. The fracture gap ended distally at 80% of the length of the femoral stem. The specimens were subsequently treated with one of the following fixation techniques: (1) cerclage band (Ic cerclage for reconstruction 8 mm; Implantcast, Buxtehude, Germany), (2) cerclage wiring using one or (3) two cerclage wires (Ø 1.6 mm, stainless steel), (4) lag screw fixation using one or (5) two cortical bone screws (Ø 3.5 mm) (Figure 1).

The cerclage band was attached and tensioned as described in the manufacturer’s operating instructions. The surgeon was advised to knot the cerclage wires with a twisted knot. The cerclage wires were twisted at least three times until the surgeon achieved sufficient tension and no spring back could be observed (Perren et al. 2011). Based on previously examined studies (Fishkin et al. 1999; Han 2000; Frisch et al. 2015), we specified the wire positioning as follows. The first wire was attached inferior to the lesser trochanter and the second one 25 mm distal to it. The screws were bicortical fixated. The first screw was placed at the level of the lesser trochanter and the second one 10 mm distal to it. This configuration was based on the senior surgeon’s experience.

The distal side of the femoral specimen was resected about 150 mm distal to the end of the implanted stem. By using a custom-designed gauge, the cut of the femoral shaft was chosen perpendicular to the Mikulicz line. The distal end of the specimen was potted in a steel sleeve (height: 10 mm, Ø: 70 mm) with a polyurethane-based fast cast resin (RenCast FC 52/53, Huntsman Advanced Materials, Basel, Switzerland).

### Biomechanical testing

**Figure 2.**
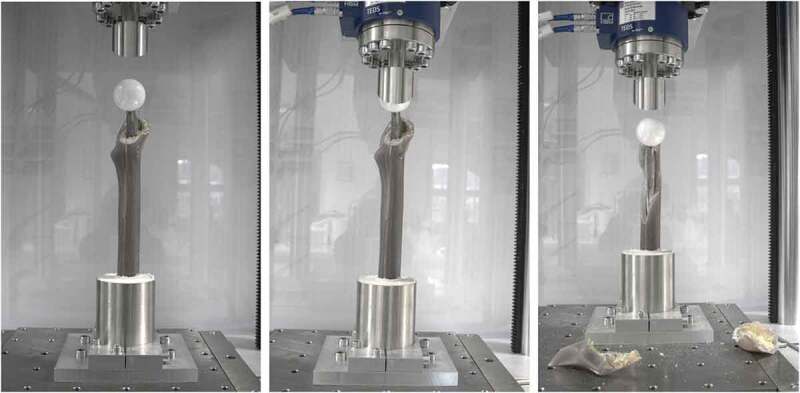
Procedure of quasi-static testing: (a) unloaded state; (b) quasi-static loading; (c) failure.

The specimens were placed in a fixture on a servo-pneumatic testing machine (Type 2082/000, DYNA-MESS Prüfsysteme GmbH, Stolberg, Germany) (Figure 2). Axial load was applied to an attached ceramic femoral head (Ø: 36 mm; size: L, Mathys AG Bettlach, Bettlach, Switzerland) through a steel stamp, replicating the acetabular cup. The potted femurs were aligned using a sliding X-Y table to ensure no transverse forces were applied to them at the beginning of the test. Based on the studies from Fishkin et al. (Fishkin et al. 1999) and Frisch et al. (Frisch et al. 2015), the quasi-static load was increased under displacement-control at a rate of 0.2 mm/s until failure load (F_failure_) was reached. F_failure_ was defined as the compressive force to initiate either audible crack, fracture of the femur, or failure of the fixation used, i.e. rupture in case of cerclage bands or cerclage wiring and screw pullout in case of lag screw fixation, respectively. Crack propagation of the IPFF or reaching the maximum load of the load cell set at 10 kN represented further failure scenarios. Similar failure modes were reported in a previous study (Frisch et al. 2015). All failure scenarios were associated with an abrupt force drop. Consequently, the failure load could be accurately determined without additional sensors such as strain gauges.

### Data analysis

A descriptive analysis and calculation of median values and ranges were carried out using MS Excel (Microsoft Corporation, Redmond, Washington, USA). The failure loads were determined as the maximum of the force curves for each test that was accompanied by a characteristic force drop after sample failure.

Inductive statistics were not considered appropriate due to the small number of samples.

## Results

**Figure 3.**
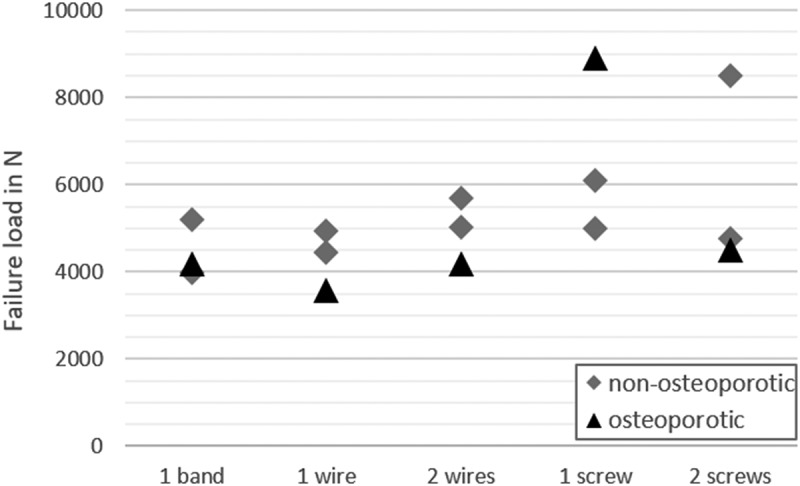
Failure loads observed for one band, one wire, two wires, one screw and two screws in non-osteoporotic (square) and osteoporotic (circle) artificial composite bones.

For cerclage band fixation, a median failure load of 4192 N with a range from 3982 N to 5189 N was determined (Figure 3), which compares with the median failure load of 4450 N ranging from 3577 N to 4927 N observed for simple cerclage fixation. The fixation technique using two cerclage wires resulted in a median failure load of 5016 N with a range from 4175 N to 5685 N. Between the cerclage fixations, no observable differences could be found. For the lag screw fixation with one screw, a median failure load of 6085 N ranging from 5000 N to 8907 N was observed, whereas two screws showed a median failure load of 4774 N with a range from 4509 N to 8502 N.

The median failure load across all tested fixation techniques was 5008 N ranging from 3982 N to 8502 N in non-osteoporotic artificial composite bones while the osteoporotic ones showed a median failure load of 4192 N with a range from 3577 N to 8907 N.

## Discussion

This pilot trial aimed to compare the biomechanical primary stability of five different fixation techniques usually used in treating IPFF. Thus, failure loads of each variant used to fix a standardised IPFF were determined in a biomechanical *in vitro* setup. The number of three samples per group was chosen as it represented a reasonable compromise between high power and low budget. Furthermore, our methodology was orientated to the study of Frisch et al. (Frisch et al. 2015), who did a similar investigation with results that are in a close range to ours. Therefore, only three specimens seemed appropriate. The influence of different bone mass densities and pore sizes, as it is similar to human specimens, was taken into account by using osteoporotic and non-osteoporotic artificial bones. Bergmann et al. have shown that several physiological loading conditions, such as single- and double-legged stance, can be simulated by the selected quasi-static load application (Bergmann et al. 2001, 2010). Due to this, this method of load application was chosen.

While Frisch et al. described mean failure loads of 4010 N in tests of double wire fixations (Frisch et al. 2015), we determined failure loads of 4585 N. Thus, our observed failure loads for cerclage wires were in similar ranges as reported in the literature but showing highly different standard deviations. The reason for this can be found in the methodology used. Although the specimen preparation and biomechanical testing were performed similarly, different implants were chosen. Frisch et al. used a standard M/L stem (Zimmer Holdings, Warsaw, Indiana) with a tapered wedge design. In contrast, the Spotorno femoral stem we used has a characteristic rib geometry. As these ribs may have caused stress peaks in the cortical replica of the artificial bone, it can potentially lead to more random failure scenarios due to material accumulation between the ribs. This could be a cause of the observed variance in failure loads.

Lenz et al. reported similar failure loads for cerclage bands and lag screw fixation systems in their biomechanical studies. For the double screw fixation, significantly higher loads were documented (Lenz et al. 2013). In our test setup, higher failure loads were determined for only individual specimens of the single and double screw fixation. This could be indicative of the potentially higher strength of the screw fixations compared to the cerclage fixation variants. However, defects are generated in the material when the screws are inserted into the artificial bone. This may have a negative effect on the fixation strength, probably leading to a higher spread of the determined failure loads.

All groups had a similar range of failure loads. However, due to the small number of samples more specific remarks regarding data distribution cannot be made. Nevertheless, contrary to our expectations, the effect of a second fixation element on achievable failure load seems to be inconclusive. Regarding crack opening, Fishkin et al. performed tests on human cadaver bones and determined a better outcome of two and three wire fixation techniques compared to the usage of one cerclage wire. This can be correlated to a higher rigidity of the fixation technique with multiple cerclage wires compared to single cerclage wiring (Fishkin et al. 1999).

Good clinical results have been documented with all treatment options (Berend et al. 2004; Ting et al. 2010; Zeh et al. 2011; Rüdiger et al. 2013).

### Conclusion

For all cerclage variants, failure first occurred at a multiple of the body mass (5.8 to 14 times) of an average adult of 70 kg. Consequently, all treatment options demonstrated adequate resistance against physiological loads (Bergmann et al. 2010) and sufficient stability for fixation of IPFF. Due to the higher than expected standard deviations within the experimental groups, further investigations with a larger sample size seem reasonable. Moreover, other fixation techniques (e.g. locking attachment plates) than those evaluated here should be focused by future studies. (Lenz et al. 2012a). Based on the results of this pilot trial, a comparative test setup using human cadaveric bones has been planned.

### Limitations

Due to ethical reasons, no human cadaveric specimens were used in this pilot trial. Besides, the usage of biomechanical artificial bones is well established in biomechanics (Brand et al. 2012; Aghayan et al. 2013; Basso et al. 2014; Schmidutz et al. 2017; Windell et al. 2020). Further, interindividual variations are ignored in artificial bones favouring standardized methodology. Nevertheless, bone density and pore size of human cancellous bone represent important bone characteristics as they highly influence its mechanical parameters. Moreover, both properties are subject to strong individual variations (Carter and Hayes 1976; Carter et al. 1977; Carter and Spengler 1978). However, as only the mechanical properties of the different fixation techniques were of interest, the aforementioned aspects were considered of little relevance to our investigation.

The cerclage wires used in this study were tightened using twist knots. Although this technique is well documented in the literature, there are other methods like the double loop techniques that have been shown to resist higher failure loads. However, none of the samples in this study failed by loosening of the knot (Roe 2002).

## Data Availability

The datasets used and analysed during the current study are available from the corresponding author on reasonable request.

## References

[cit0001] Aghayan S, Shepherd DET, Davis ET. 2013. A biomechanical study of the Birmingham mid head resection arthroplasty: effect of stem size on femoral neck fracture. Proc Inst Mech Eng H. 227(8):913–918. eng. doi:10.1177/0954411913485057. 23636766

[cit0002] Angelini A, Battiato C. 2015. Past and present of the use of cerclage wires in orthopedics. Eur J Orthop Surg Traumatol. 25(4):623–635. eng. doi:10.1007/s00590-014-1520-2. 25186972

[cit0003] Apivatthakakul T, Phaliphot J, Leuvitoonvechkit S. 2013. Percutaneous cerclage wiring, does it disrupt femoral blood supply? A cadaveric injection study. Injury. 44(2):168–174. eng. doi:10.1016/j.injury.2012.10.016. 23164676

[cit0004] Basso T, Klaksvik J, Syversen U, Foss OA. 2014. A biomechanical comparison of composite femurs and cadaver femurs used in experiments on operated Hip fractures. J Biomech. 47(16):3898–3902. eng. doi:10.1016/j.jbiomech.2014.10.025. 25468304

[cit0005] Berend KR, Lombardi AV, Mallory TH, Chonko DJ, Dodds KL, Adams JB. 2004. Cerclage wires or cables for the management of intraoperative fracture associated with a cementless, tapered femoral prosthesis: results at 2 to 16 years. J Arthroplasty. 19(7):17–21. eng. doi:10.1016/j.arth.2004.06.008. 15457413

[cit0006] Bergmann G, Deuretzbacher G, Heller M, Graichen F, Rohlmann A, Strauss J, Duda G. 2001. Hip contact forces and gait patterns from routine activities. J Biomech. 34(7):859–871. doi:10.1016/S0021-9290(01)00040-9. 11410170

[cit0007] Bergmann G, Graichen F, Rohlmann A, Bender A, Heinlein B, Duda GN, Heller MO, Morlock MM. 2010. Realistic loads for testing Hip implants. Biomed Mater Eng. 20(2):65–75. eng. doi:10.3233/BME-2010-0616. 20592444

[cit0008] Berry DJ. 1999. Epidemiology: hip and Knee. Orthop Clin North Am. 30(2):183–190. doi:10.1016/s0030-5898(05)70073-0. 10196420

[cit0009] Brand S, Klotz J, Hassel T, Petri M, Haasper C, Bach F-W, Krettek C, Goesling T. 2012. Intraprosthetic fixation techniques in the treatment of periprosthetic fractures-A biomechanical study. World J Orthop. eng, 3:162–166. doi:10.5312/wjo.v3.i10.162. 23326763 PMC3536858

[cit0010] Capone A. 2017. Periprosthetic fractures: epidemiology and current treatment. Clin Cases Miner Bone Metab. 14(2):189–196. eng. doi:10.11138/ccmbm/2017.14.1.189. 29263732 PMC5726208

[cit0011] Carter DR, Hayes WC. 1976. Bone compressive strength: the influence of density and strain rate. Science. 194(4270):1174–1176. eng. doi:10.1126/science.996549. 996549

[cit0012] Carter DR, Hayes WC, Carter DR. 1977. The compressive behavior of bone as a two-phase porous structure. J Bone Joint Surg Am. 59(7):954–962. eng. doi:10.2106/00004623-197759070-00021. 561786

[cit0013] Carter DR, Spengler DM. 1978. Mechanical properties and composition of cortical bone. Clin Orthop Relat Res 135:192–217. eng. 361320

[cit0014] Della Rocca GJ, Leung KS, Pape H-C. 2011. Periprosthetic fractures: epidemiology and future projections. J Orthop Trauma. 194(Supplement 2):S66–70. eng. doi:10.1126/science.996549. 21566478

[cit0015] Ferbert T, Jaber A, Gress N, Schmidmaier G, Gotterbarm T, Merle C. 2020. Impact of intraoperative femoral fractures in primary Hip arthroplasty: a comparative study with a mid-term follow-up. Hip Int. 30(5):544–551. eng. doi:10.1177/1120700019849911. 31096789

[cit0016] Fishkin Z, Han S-M, Ziv I. 1999. Cerclage wiring technique after proximal femoral fracture in total Hip arthroplasty. J Arthroplasty. 14(1):98–101. doi:10.1016/S0883-5403(99)90209-7. 9926960

[cit0017] Frisch NB, Charters MA, Sikora-Klak J, Banglmaier RF, Oravec DJ, Silverton CD. 2015. Intraoperative periprosthetic femur fracture: a biomechanical analysis of cerclage fixation. J Arthroplasty. 30(8):1449–1457. Epub 2015 Feb 28. eng . doi:10.1016/S0883-5403(99)90209-7. 25765131

[cit0018] Han SM. 2000. Comparison of wiring techniques for bone fracture fixation in total hip arthroplasty. Tohoku J Exp Med. 192(1):41–48. doi:10.1620/tjem.192.41. 11128867

[cit0019] Horner D, Pandor A, Goodacre S, Clowes M, Hunt BJ. 2019. Individual risk factors predictive of venous thromboembolism in patients with temporary lower limb immobilization due to injury: a systematic review. J Thromb Haemost. 17(2):329–344. eng. doi:10.1111/jth.14367. 30580466 PMC6392108

[cit0020] Lenz M, Perren SM, Gueorguiev B, Höntzsch D, Windolf M. 2013. Mechanical behavior of fixation components for periprosthetic fracture surgery. Clinical Biomechanics. 28(9–10):988–993. eng. doi:10.1016/S0883-5403(99)90209-7. 24080369

[cit0021] Lenz M, Perren SM, Gueorguiev B, Richards RG, Krause F, Fernandez Dell’Oca A, Höntzsch D, Windolf M. 2012a. Underneath the cerclage: an ex vivo study on the cerclage-bone interface mechanics. Arch Orthop Trauma Surg. 132(10):1467–1472. Epub 2012 Jun 28. eng . doi:10.1016/j.arth.2015.02.026. 22740062

[cit0022] Liu B, Ma W, Li H, Wu T, Huo J, Han Y. 2019. Incidence, classification, and risk factors for intraoperative periprosthetic femoral fractures in patients undergoing total hip arthroplasty with a single stem: a retrospective study. J Arthroplasty. 34(7):1400–1411. eng. doi:10.1016/j.arth.2015.02.026. 30956049

[cit0023] Masri BA, Meek RMD, Duncan CP. 2004. Periprosthetic fractures evaluation and treatment. Clin Orthop Relat Res. 80–95. eng. doi:10.1097/00003086-200403000-00012 15057082

[cit0024] McCulloch RS, Roe SC, Marcellin-Little DJ, Mente PL. 2012. Resistance to subsidence of an uncemented femoral stem after cerclage wiring of a fissure. Vet Surg. 41:163–167. Epub 2011 Jul 19. eng . doi:10.1111/j.1532-950X.2011.00858.x. 21770982

[cit0025] OECD. 2019. Health at a Glance 2019. OECD Indicators. Paris:OECD.

[cit0026] Perren SM, Fernandez Dell’Oca A, Lenz M, Windolf M. 2011. Cerclage, evolution and potential of a Cinderella technology. An overview with reference to periprosthetic fractures. Acta Chir Orthop Traumatol Cech. 78: 190–199. eng. 21729634

[cit0027] Roe SC. 2002. Evaluation of tension obtained by use of three knots for tying cerclage wires by surgeons of various abilities and experience. J Am Vet Med Assoc. eng, 220:334–336. doi:10.2460/javma.2002.220.334. 11829264

[cit0028] Rüdiger HA, Betz M, Zingg PO, McManus J, Dora CF. 2013. Outcome after proximal femoral fractures during primary total hip replacement by the direct anterior approach. Arch Orthop Trauma Surg. 133:569–573. Epub 2013 Feb 19. eng . doi:10.1007/s00402-013-1697-6. 23420064

[cit0029] Schmidutz F, Woiczinski M, Kistler M, Schröder C, Jansson V, Fottner A. 2017. Influence of different sizes of composite femora on the biomechanical behavior of cementless hip prosthesis. Clin Biomech (BristolAvon). eng, 41:60–65. doi:10.1016/j.clinbiomech.2016.12.003. 27960138

[cit0030] Ting NT, Wera GD, Levine BR, Della Valle CJ. 2010. Early experience with a novel nonmetallic cable in reconstructive hip surgery. Clin Orthop Relat Res. eng, 468:2382–2386. doi:10.1007/s11999-010-1284-x. 20204557 PMC2919859

[cit0031] Watts CD, Abdel MP, Lewallen DG, Berry DJ, Hanssen AD. 2015. Increased risk of periprosthetic femur fractures associated with a unique cementless stem design. Clin Orthop Relat Res. eng, 473:2045–2053. doi:10.1007/s11999-014-4077-9. 25502478 PMC4419010

[cit0032] Windell L, Kulkarni A, Alabort E, Barba D, Reed R, Singh HP. 2020. Biomechanical comparison of periprosthetic femoral fracture risk in the Exeter V40, CPT, and dePuy C-Stem in a sawbone model. J Arthroplasty. Eng. doi:10.1016/j.arth.2020.07.061 32826144

[cit0033] Zeh A, Radetzki F, Diers V, Bach D, Röllinghoff M, Delank KS. 2011. Is there an increased stem migration or compromised osteointegration of the mayo short-stemmed prosthesis following cerclage wiring of an intrasurgical periprosthetic fracture? Arch Orthop Trauma Surg. 131:1717–1722. Epub 2011 Jun 29. eng . doi:10.1007/s00402-011-1342-1. 21713540

[cit0034] Zhao R, Cai H, Liu Y, Tian H, Zhang K, Liu Z. 2017. Risk factors for intraoperative proximal femoral fracture during primary cementless THA. Orthopedics. eng, 40:e281–e287. doi:10.3928/01477447-20161116-06. 27874909

